# The Rationale for the Intra-Articular Administration of Clodronate in Osteoarthritis

**DOI:** 10.3390/ijms22052693

**Published:** 2021-03-07

**Authors:** Antimo Moretti, Marco Paoletta, Sara Liguori, Walter Ilardi, Francesco Snichelotto, Giuseppe Toro, Francesca Gimigliano, Giovanni Iolascon

**Affiliations:** 1 Department of Medical and Surgical Specialties and Dentistry, University of Campania “Luigi Vanvitelli”, 81100 Naples, Italy; antimo.moretti@unicampania.it (A.M.); marco.paoletta@unicampania.it (M.P.); sara.liguori@unicampania.it (S.L.); walter.ilardi@studenti.unicampania.it (W.I.); francesco.snichelotto@studenti.unicampania.it (F.S.); giuseppe.toro@unicampania.it (G.T.); 2 Department of Mental and Physical Health and Preventive Medicine, University of Campania Luigi Vanvitelli, 81100 Naples, Italy; francesca.gimigliano@unicampania.it

**Keywords:** clodronic acid, clodronate, osteoarthritis, injections, intra-articular, diphosphonates

## Abstract

Background: Several pharmacological therapeutic approaches have been proposed to manage osteoarthritis (OA), including intra-articular (IA) injections. Although the discovery of clodronate, a bisphosphonate, dates back to the 1960s and the effects of its IA administration have been investigated for decades in animal models, mechanisms of action of this drug are not quite clear, particularly in OA. This scoping review is an overview of the biological as well as the clinical role of clodronic acid in OA. Method: A scoping review based on the PRISMA-ScR (Preferred Reporting Items for Systematic Reviews and Meta-Analyses Extension for Scoping Reviews) model was performed to characterize the mechanisms of action of IA clodronate in OA and to evaluate its efficacy from a clinical point of view. Results: Several effects of clodronate have been observed in animal models of OA, including depletion of synovial lining cells that results in reduced production of chemokines (IL-1, TNF- α), growth factors (TGF-β, BMP 2/4), and metalloproteases (MMP 2/3/9); prevention of cartilage damage, synovial hyperplasia, and proteoglycans loss; reduction in joint inflammation, joint swelling, and osteophyte formation. From a clinical perspective, patients with knee OA treated with IA clodronate experienced improvements in pain and joint mobility. Conclusion: Clodronate appears to have different mechanisms of action interfering with the pathogenic processes contributing to OA development and progression. This intervention demonstrated positive effects for patients affected by knee OA.

## 1. Introduction

Osteoarthritis (OA) is a chronic degenerative joint disease with a significant impact on the quality of life. Osteoarthritis is consistently ranked among the leading contributors to disability, accounting for 2% of the total global years lived with disability [1]. This condition commonly affects the knee, hip, hand, and spine, but all joints can be involved. From a pathophysiological perspective, OA is characterized by impaired metabolism of joint tissues leading to structural changes, such as cartilage degradation, subchondral bone remodeling, and osteophyte formation [2]. The main clinical complaints of OA patients are pain and functional limitation [3]. Radiological assessment allows the typical findings of OA to be revealed, such as joint space narrowing, osteophytes, subchondral cysts, and sclerosis that are commonly assessed by Kellgren and Lawrence (KL) scale [4]. Several guidelines for the management of OA recommend a multimodal approach, including pharmacological and non-pharmacological interventions, such as therapeutic exercise, weight management, topical and oral non-steroidal anti-inflammatory drugs (NSAIDs), as well as intra-articular (IA) injections of different agents [5,6,7]. However, evidence of this latter approach is conflicting. Intra-articular glucocorticoid injections are recommended for patients with knee and/or hip OA [5], although it seems to accelerate joint degeneration [8], whereas a local administration of other IA agents, such as hyaluronic acid (HA), is still debated [5]. Intra-articular administration of clodronate, a first-generation non-nitrogenous bisphosphonate (BP), has been provided to patients with OA, although poor evidence supports this practice. It seems that clodronate might be useful in managing OA, based on its anti-erosive, anti-inflammatory, and analgesic effects. Available literature focuses on different administration routes of clodronate for treating OA. In this context, the IA route has been poorly investigated [9]. It has been hypothesized that IA clodronate administration may promote combined effects on joint tissues, including suppression of local bone turnover, inhibition of local release of pro-inflammatory cytokines, and consequent nociceptors stimulation [10]. However, these evidences mainly derived from animal models where clodronate is administered in liposomal form, which seems to have a greater effect on local phagocytic cells compared to the free formulation [11].

This scoping review aims to summarize current knowledge about the role of IA clodronate administration in the treatment of OA.

## 2. Materials and Methods

According to the PRISMA-ScR (Preferred Reporting Items for Systematic Reviews and Meta-Analyses Extension for Scoping Reviews) model, we performed a scoping review [12] by involving a technical expert panel (TEP) that consisted of 8 physicians (five pain rehabilitation specialists—GI, FG, AM, WI, FS, two experts in scoping review methodology—MP, SL, and one orthopedic surgeon—GT). The TEP evaluated the rationale for the use of IA clodronate in OA, particularly focusing on its mechanisms of action.

### 2.1. Search Strategy

The TEP planned a search on PubMed (Public MedLine, run by the National Center of Biotechnology Information, NCBI, of the National Library of Medicine of Bethesda, Bethesda, MD, USA), with ad-hoc search strings with selected key words also used as MeSh (Medical Subject Headings) terms: osteoarthritis, IA injection, clodronic acid (Table 1).

### 2.2. Study Selection

According to the study objective, the TEP defined the characteristics of the sources of evidence, considering for eligibility any research published until 31 October 2020 and including only those in the English language. Eligibility criteria (inclusion/exclusion) are reported in Table 2.

### 2.3. Data Extraction and Quality Assessment

Clinical research, interventional (randomized or non-randomized controlled clinical trials) and observational studies, case series or case reports, and animal studies meeting our eligibility criteria were selected. Results and findings from each included study were qualitatively analyzed.

## 3. Results

**Figure 1:**
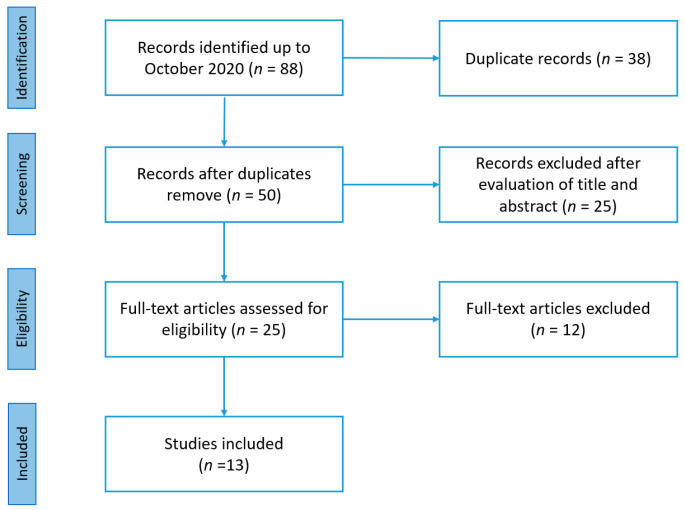
PRISMA-ScR (Preferred Reporting Items for Systematic Reviews and Meta-Analyses Extension for Scoping Reviews) flow-chart for study selection.

Eighty-eight items were found. After duplicate removal, fifty records remained; they were screened based on titles and abstracts for inclusion/exclusion criteria, and twenty-five studies were excluded. After full text reading, twelve of them were excluded because did not fulfill our inclusion criteria (Table 2). Finally, thirteen articles published between 1993 and 2020 were selected, ten preclinical studies referring to different animal models and three clinical studies (Figure 1).

Table 3 presents the characteristics and main findings of the included studies.

### 3.1. Animal Studies

In the present scoping review, we included ten preclinical studies investigating potential direct and indirect effects of IA clodronate in animal models of OA (7 mice, 2 rabbits, 1 sheep).

Van Lent et al. evaluated clodronic acid effects on synovial lining cells in mice [13]. Authors administrated a single IA injection of liposomes encapsulating clodronate in the knee joint. They found a progressive reduction in the synovial cells starting from the third day after the injection, which was at its maximum seven days later. From the ninth day after the injection, a partial recovery of the cellular population of the lining layer was found. Thirty days later, the recovery of the lining layer had reached 60% compared to the treatment initiation. No effect was found on fibroblasts (synoviocytes type B). Moreover, the authors investigated the effects of clodronate on proteoglycan synthesis and degradation by measuring the ^35^Sulfur uptake and loss from the patella. At day 2 after IA injection, a low but significant inhibition of proteoglycan synthesis and a setting up of 10–20% of proteoglycan degradation were observed. However, at day 3, proteoglycans metabolism went back to normal levels. In addition, the authors induced an experimental arthritis by injecting hen egg lysozyme coupled to poly-L-lysine into the knee joints of mice that previously had received polyclonal antibodies directed against lysozyme. They found that clodronate prevented experimental arthritis assessing 99 mTC uptake, which was significantly lower in the treated group. Overall, these findings suggest that clodronate downregulates joint inflammation, thereby preventing cartilage damage by removing a key source of macrophagic chemotactic factors.

In the same year, Van Lent et al. published a further study about the role of clodronate on phagocytic synovial lining cells in the induction of experimental arthritis [14]. They compared a group of mice treated with a single IA injection of liposomes containing clodronate to another group receiving empty liposomes. After 7 days, they induced an experimental immune-complex mediated arthritis by injecting the cationic antigen lysozyme coupled to poly-L-lysine (PLL) in knee joints of mice that were previously treated with polyclonal antibodies directed against lysozyme. In the clodronate group, they found less intense joint alterations than in the control group. Indeed, in the intervention group, there was a reduction in joint swelling, IA effusion, and inflammatory infiltrate (neutrophils), chemokine and chemotactic factors (IL-1, TNF-α), decrease in proteoglycan degradation (27 ± 9% vs. 47 ± 10%; *p* < 0.05), and inhibition of proteoglycan synthesis (21 ± 9% vs. 41 ± 11%; *p* < 0.05) compared to controls. Finally, the authors found that clodronate liposomes did not damage synovial blood vessels evaluated by the migration of inflammatory cells of Complement component 5a (C5a). The authors claimed that liposome containing clodronate might interfere with the arthritic process by blocking inflammatory cell influx by selectively eliminating phagocytic cells. Some years later, Van Lent et al. investigated the impact of IA clodronate on cartilage damage in mice models of induced arthritis [15]. To produce joint damage, they first immunized the mice by subcutaneous administration of bovine collagen type II emulsified in Freund’s complete adjuvant, and after 21 days, they administered an intraperitoneal injection of type II collagen. Thus, development of polyarthritis was observed between 4 and 5 weeks later. They administered clodronate-laden liposome suspension in the right knee and empty liposome in the left knee 7 days before inducing arthritis. The authors observed no difference between the groups for proteoglycan loss. On the other hand, chondrocyte depletion 12 days after the injection was significantly lower in the clodronate group both in the femoral (70% protection) and in the patellar (60% protection) cartilage layers.

Highton et al. evaluated the effects of clodronate-liposomes on macrophages in a sheep model of arthritis [16]. They analyzed three groups: group 1 treated with clodronate liposomes, group 2 with saline liposomes, and group 3 untreated. In the first two groups, autoimmune arthritis was induced 7 days before intervention. To induce the arthritic process, they previously immunized the sheep by subcutaneous administration of ovalbumin in Freund’s complete adjuvant and subsequently performed an IA injection of 5 mg ovalbumin in 0.5 mL saline in the right hock joint. The authors found no significant difference between arthritis-induced groups in terms of joint swelling, measured in anterior–posterior hock joint diameter. However, joint swelling was significantly worse in both groups in comparison to non-arthritic sheep. The histological score measured considering synovial membrane thickness, the intensity of inflammation, inflammatory infiltrate, and extent of fibrin deposition were not significantly different between groups 1 and 2, but it was worse for these 2 groups than for the third non-arthritic group. Ceponis et al. investigated the long-term effects of IA liposomal clodronate in antigen-induced arthritis (AIA) in rabbits [17]. Monoarthritis was induced by injecting 5 mg of ovalbumin solution into the knee joint after immunization obtained by subcutaneous administration of ovalbumin and Freund’s complete adjuvant. They divided 17 rabbits into two groups: group 1, including 10 rabbits, treated with clodronate, and group 2, consisting of 7 specimens, treated with empty liposomes. Weekly injections were performed for both groups for seven weeks. The authors found a statistically significant reduction (*p* < 0.05) of joint diameter after three IA injections of the liposomal clodronate compared to controls. Moreover, no significant between-group difference was observed in terms of serum C-Reactive Protein (CRP) levels before arthritis induction and at the end of the experiment. In the same study, an arbitrary radiological joint destruction score, based on pathological changes, such as osteophyte formation and osteosclerosis, was applied by an expert radiologist. This score was significantly lower in the liposome clodronate group than empty liposome group at 2 weeks (0 vs. 0.29 ± 0.49; *p* < 0.05) and at 4 weeks (0 vs. 0.57 ± 0.79; *p* < 0.05) from arthritis induction, suggesting a protective role of clodronate on radiographic findings of OA. Considering histological analysis, significant between-group differences were observed in terms of reduction in synovial lining cell hyperplasia score (1.7 ± 0.48 vs. 2.4+/−0.79; *p* < 0.05), macrophage density RAM-11+ (618 ± 166 vs. 898 ± 179 cells/mm^2^; *p* < 0.05), and matrix-bound solubilized TNFα (0.9 ± 0.95 vs. 2.7 ± 1.2 *p* < 0.05). Moreover, the clodronate group showed a significant thickness reduction only for the superficial cartilage layer (36%; *p* = 0.022).

Bakker et al. evaluated the role of the overexpression of TGF-β in the development of OA in mice [18]. They found that high levels of active TGF-β1 in knee joint produced by the synovial lining cells were associated with specific histopathological changes, such as the hyperplasia of synovium and chondro-osteophyte formation. Administration of a single IA injection of clodronate in mice knees resulted in the depletion of synovial lining cells along with a reduction in TGF-β-related alterations, including chondro-osteophyte formation and accumulation of extracellular matrix in synovium at 7 days.

Blom et al. evaluated the role of synovial macrophages in the formation of osteophytes and other typical changes in OA in mice [19]. An IA knee injection of clodronate was compared to that of empty liposomes in arthritis-induced models (two IA injections of collagenase on alternate days into murine knee joints). At the histological level, a significant decrease in osteophytes size and lower calcification of the cartilage at 7 (84 ± 16%) and 14 (66 ± 13%) days were reported in the clodronate group. Moreover, significant reduction in fibrosis (*p* < 0.05) and inflammatory cell infiltrate (*p* < 0.02) were also found at the same time-points. The authors reported a lower expression of a macrophage activation marker (MRP14) in the superficial synovial layer in mice treated with clodronate at day 14 (*p* < 0.05). They found similar results in the expression of BMP-2 (0.3 ± 0.3 vs. 1.0 ± 0.06; *p* < 0.005), BMP-4 (0.1 ± 0.2 vs.1.0 ± 0.8; *p* < 0.05), and TGF-β (0.8 ± 0.3 vs. 2.2 ± 0.05; *p* < 0.05) in the treated group compared to controls.

Gomez-Barrena et al. evaluated the effects of IA liposomal clodronate on levels of cartilage oligomeric matrix protein (COMP) in joint tissues in an experimental model of arthritis in rabbits [20]. Monoarthritis was induced by injecting 5 mg of ovalbumin into the knee joint after the immunization was obtained by multiple subcutaneous administration of ovalbumin and Freund’s complete adjuvant. After the induction of arthritis, researchers performed seven weekly IA injections of liposomal clodronate in one group, empty liposomes in a second group, and saline injection into the other knee as controls. Seventy-two hours after the last injection, chondrocyte densities did not differ between the groups, while COMP staining of the superficial layer of cartilage was well preserved in the clodronate group compared to the empty liposome group (*p* = 0.043). Moreover, COMP staining of the middle and deep layer of cartilage had significantly increased results in the clodronate group compared to healthy control joints (*p* = 0.027 and 0.017, respectively).

Blom et al. investigated the role of synovial macrophages and matrix metalloproteinases (MMP) in early and late OA in mice [21]. The intervention group was treated with a single IA injection of clodronate-containing liposomes, while the control group received an empty liposome IA injection. After seven days from induced OA (IA injection of collagenase), the synovial expressions of MMP-2, MMP-3, and MMP-9 were inhibited in the clodronate group and upregulated in control joints. At 14 days, the authors found a significant increase in the MMP-generated neoepitope (VDIPEN) in the control group (*p* < 0.001) that was absent in the clodronate treated group. No between-group difference in the cartilage expression of MMPs was observed. These findings corroborated the selectivity of clodronate for synovial tissue in animal models of OA.

Sun et al. evaluated the role of macrophages in the progression of induced OA in obese mice [22]. They fed a group of mice a specific high fat diet and then induced OA by dissecting the medial meniscus of the left knee. The authors performed three IA injections of clodronate liposomes or empty liposomes: the first injection one week before OA induction, and the other two injections at one and eight weeks. Histological analysis showed a significant reduction in the number of synovial macrophages (*p* < 0.05) and synovium thickness (*p* < 0.05) in the clodronate group. In addition, increased proteoglycans content, reduced OA severity according to the Mankin score (*p* < 0.05), and expression of collagen type 10 (COL10) (*p* < 0.05), DIPEN (*p* < 0.05), and NITEGE (*p* < 0.05) were observed in the clodronate treated group compared to controls.

### 3.2. Clinical Studies

Rossini et al. compared symptomatic and functional benefits of patients with severe knee OA (KOA) treated with IA injections of HA or clodronate at three different doses [23]. The authors enrolled 150 patients, randomly allocated to receive only one of five possible treatment regimens. Participants were evaluated for pain (VAS), joint mobility, Lequesne index, and need for rescue therapy (acetaminophen). They found that IA clodronate provides significant symptomatic and functional improvements similar to those obtained by IA HA. However, no significant differences were detected among the four clodronate treatment regimens (0.5 mg, 1 mg, 2 mg, 1 + 1 mg) according to both Lequesne score and VAS, except for a significant linear trend for a dose–response for pain during active movement.

Palmieri et al. evaluated the effects of a cross-linked HA IA injection, alone or in combination with diclofenac sodium or sodium clodronate, for managing KOA-related pain [24]. They included 62 patients with symptomatic bilateral medial tibiofemoral KOA (K–L grade II and III), dividing them into three groups. Group 1 received one injection of HA alone, group 2, a single injection of HA plus diclofenac sodium, and group 3, one injection of HA plus sodium clodronate. Even if all groups showed statistically significant pain relief at both the 3- and 6-month follow-ups, the combined treatment with HA and clodronate was significantly more effective when compared with the other two groups.

In another study, Rossini et al. investigated the efficacy and tolerability of IA clodronate compared to saline solution in patients with symptomatic KOA [10]. Eighty patients received IA injections of 2 mg clodronate once weekly or placebo for 4 weeks with further 6 fortnightly follow-up visits. The clodronate group reported statistically significant greater benefits compared to patients receiving saline solution injections in terms of VAS, Western Ontario and McMaster University (WOMAC) scale, and Lequesne Index.

## 4. Discussion

**Figure 2:**
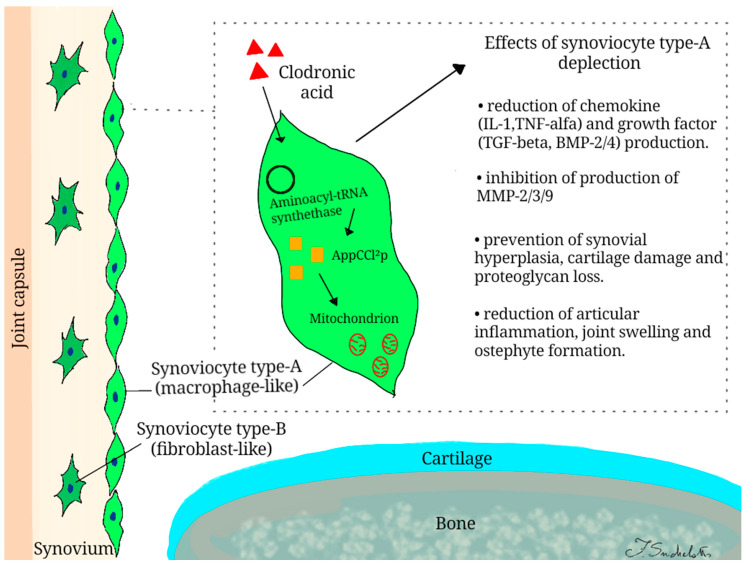
Mechanisms of action of clodronic acid in intra-articular environment.

Our study summarizes the mechanisms of action of clodronate, administered via the IA route, as well as the clinical implications of this intervention in the treatment of OA (Figure 2). In general, different effects of this drug have been reported in the joint environment, particularly targeting synovial tissue, with indirect effects on articular cartilage.

Clodronate is a non-nitrogenous BP that exhibits a unique mechanism of action. Once internalized by macrophage cells, it is metabolized to AppCCl2p, a non-hydrolysable analog of ATP, by aminoacyl-tRNA synthetase [25]. This metabolite accumulates inside the cell and exerts a series of damaging effects, especially at the mitochondrial level, leading to cell apoptosis [26]. After IA injection, clodronate is phagocytosed by type A synoviocytes (macrophage-like cells) that are involved in the pathogenesis of OA [13,19,27]. A selective depletion of these cells by clodronate, that did not affect type B synoviocytes (fibroblast-like cells) has been observed, as demonstrated both in vivo and in vitro [13,27]. The reduction in type A synoviocytes might be useful in preventing the development of OA.

Clodronate IA injection reduces chemical mediators involved in the development of inflammation (IL-1, TNFα) as well as molecules involved in structural alterations occurring in OA (TGFβ, BMP-2, and BMP-4) [14,17,18,19]. The anti-inflammatory effect is probably due to the clodronate metabolite, which can alter the DNA binding capacity of the transcription factor NF-κB [28]. Otherwise, a reduction in IA levels of TGFβ has been shown to prevent typical structural alterations affecting joints in OA (chondro-osteophyte formation and accumulation of extracellular matrix in synovium) [18]. Finally, the reduction in BMP-2/4 is crucial in the formation of osteophytes, as demonstrated by the stimulation of chondrogenesis and bone formation through their injection into the knee joint [19,29,30].

Furthermore, the apoptosis of macrophages obtained by IA clodronate injection might result in reduced levels of enzymes that have detrimental effects on the joint tissues (MMP-2, MMP-3, MMP-9). A key role in the development of OA seems to be played by MMP-3, which is particularly overexpressed in animal models of OA [31]. Blom et al. investigated this process by studying knocked-out mice for MMP-3 that develop OA-like changes only in the superficial layer of synovium and not in the deep layer of the cartilage tissue [13,14,15,19,21]. This is probably due to the distribution of type A synoviocytes only in the surface layer [27]. In the experimental forms of arthritis, the IA administration of clodronate seems to prevent the development of synovial hyperplasia, as demonstrated by the reduction in synovial activation markers, such as the number of RAM-11+ macrophages and the MRP8/14 expression [17,19,22].

The use of clodronate seemed to determine positive effects in other tissues besides the synovial one by modulating the expression of other markers of joint damage. COMP is a component of the cartilage matrix contributing to the structural integrity of the joint by forming bridges between collagen type II and IX, and it has been proposed as a marker of disease activity in OA. Clodronate modulates COMP expression in two ways. During synovitis, as a sign of damage, high COMP levels can be observed. Clodronate reduces COMP levels in synovial cells but simultaneously maintains or even increases COMP levels in hyaline articular cartilage by modulating gene transcription of this protein in chondrocytes [20]. Another study investigated the role of clodronate as a potential chondroprotective agent, as suggested by changes in gene expression of mice with a high-fat diet (HFD)-induced OA. In these animal models, overexpression of genes encoding for biochemical markers of OA progression and severity (COL10, DIPEN, and NITEGE) were reduced by clodronate IA administration [22]. The absence of synovial macrophages in clodronate-treated OA mice on HFD resulted in an increased content of proteoglycan compared with untreated OA mice fed with HFD. In this context, the role of diet should be better investigated [22]. It has been demonstrated that clodronate-liposome reduces proteoglycan synthesis after IA injection for two days, but no further increase in proteoglycan degradation was observed. These actions might be associated with histopathological severity of OA (i.e., lower Mankin score) and the reduction in knee joint swelling, as demonstrated in clodronate-treated mice [13,22].

The crucial role of macrophages in the pathogenesis of OA has been further confirmed by the reduction in osteophytes and fibrosis as well as lining thickening and influx of inflammatory cells induced by phagocytic cell depletion following clodronate injections [19].

From a clinical perspective, patients with KOA treated with IA clodronate showed significant pain relief and functional improvement compared to those receiving saline injection [10]. Further evidence investigated the efficacy of IA administration of clodronate at different doses and compared it with HA. Clinical findings suggest that clodronate is as effective as HA in patients with KOA, with statistically and clinically meaningful progressive improvement in pain and function lasting at least 2 weeks after the last injection without a dose–response relationship, except for a significant linear trend for pain relief during active movement [23]. Clodronate injections have also been administered in combination with HA in a preliminary study, demonstrating greater pain relief compared to HA plus diclofenac or HA alone at both the 3- and 6-month follow-up [24].

The ability of clodronate to reduce pain in OA could be due to the modulation of multiple pain generators not only in the intra-articular environment [32]. In chronic pain conditions, clodronate blocks the release of ATP at the vesicular level by interfering with the purinergic chemical transmission [33]. More in detail, clodronate appears to act as a ketone body analog by allosterically inhibiting the vesicular nucleotide transporter (VNUT) that facilitates vesicular ATP storage. Vesicular ATP release from the primary afferent sensory nerves to the spinal cord is a key mechanism in the development of chronic pain. Consequently, inhibition of the VNUT can be useful also for chronic pain associated with OA [34]. It should be underlined that clodronate dosages used in clinical studies for IA treatment of OA are very low to justify systemic effects on pain modulation mechanisms.

It has been hypothesized that BPs also exert antiangiogenic effects by inhibiting the proliferation of human endothelial cells and reducing the expression of basic fibroblast growth factor and vascular endothelial growth factor [35]. Once IA is administered, BPs could reduce the osteochondral neurovascular invasion observed in OA, thus counteracting the sensitization of sensory neural components of joints to mechanical stress [36].

Moreover, the subchondral bone in OA is commonly affected by the localized reduction in bone mass due to increased bone turnover as well as by histological microfractures that are seen by MRI in many patients with OA [37,38]. The inhibition of osteoclast activity by BPs not only reduces bone turnover but also leads to a reduction in extracellular acidity. This latter mechanism reduces the activation of primary nociceptive bone afferents mediated by the ion channels ASICs (acid-sensing ion channels) and TRPV1 (transient receptor potential cation channel subfamily V member 1) sensitive to extracellular acidity [32,39,40].

Another topic that deserves to be properly addressed in the context of the mechanism of action of clodronate IA injections in OA is the reduction in bone marrow lesions (BMLs) that are associated with pain and progression in joint damage [41,42]. However, it should be underlined that evidence of the role of IA clodronate in the management of BMLs in OA is scant. BMLs are Magnetic Resonance Imaging (MRI) findings, especially affecting subchondral bone. Changes in BML size and MRI signal intensity occur faster than cartilage alterations considering the subchondral bone’s high adaptive capacity to biomechanical stimuli [41]. In this site, micro-edema, microfracture, subchondral cyst, as well as increased bone turnover and angiogenesis, are commonly observed. Evidence supports the positive association between BMLs and pain in OA [43], whereas the pathogenic role of BMLs in the progression of OA is still debated. The rationale for using BPs in BMLs is mainly based on inhibition of osteoclast activity. It has been supposed that by reducing bone resorption, structural failure could be avoided, but clinical studies are controversial [43,44]. On the other side, some studies investigating the role of BPs on early OA suggest promising results in terms of pain relief and reduction in BMLs size [45,46].

There are still unclear points regarding how much the efficacy of clodronate is linked to local effects resulting from the contact of the drug with cells and joint tissues and how much to the passage into the circulation of a portion of the molecule that would exert similar effects to those of traditional drug administration routes. However, considering the very low dosages used for IA delivery of clodronate compared to those administered for oral or parenteral therapy, a systemic effect of this drug after IA injection is unlikely.

Finally, it should be underlined that studies included in our review are quite heterogeneous, particularly in terms of the type of animal models and methods of experimental-induced joint damage. Moreover, no translational research has been found, thus not allowing us to advance some considerations about the clinical implications of the structural changes observed in preclinical studies.

## 5. Conclusions

According to available evidence, the rationale for the IA administration of clodronate is based on its effects on different pathogenic mechanisms of OA, particularly in terms of phagocytic synovial lining cell depletion that result in reduced production of pro-inflammatory mediators with consequent prevention of events characterizing articular cartilage damage. Most of these findings were drawn from animal models, while their translational applications in clinical settings were poorly investigated. Nevertheless, in this context, IA clodronate seems to be effective for pain relief and functional improvement in patients affected by KOA.

## Figures and Tables

**Table 1:** Search strategy.

(“Clodronic Acid”[Mesh]) AND “Osteoarthritis”[Mesh]
(“Clodronic Acid”[Mesh]) AND “Injections, Intra-Articular”[Mesh]
((“Clodronic Acid”[Mesh]) AND “Osteoarthritis”[Mesh]) AND “Injections, Intra-Articular”[Mesh]
Osteoarthritis AND Clodronic acid
Intra-articular injection AND Clodronic acid

**Table 2:** Eligibility criteria.

Eligibility Criteria
*Inclusion criteria:* - Animal studies in physiological condition. - Animal models of induced osteoarthritis. - Interventional and observational clinical studies
*Exclusion criteria:* - Review articles. - Conference abstracts and editorials. - In vitro studies. - Studies about rheumatoid arthritis or acute arthritis not resembling osteoarthritis (e.g., traumatic intra-articular fracture induced arthritis) * - Studies about clodronic acid with different routes of administration.

* Although only OA was included for clinical trials, animal models that were initially inflammatory but produced secondary OA were permitted.

**Table 3:** Characteristics and main findings of the included studies.

Authors	Study Design	Site	Administration Route	Sample Size	Main Findings
Van Lent et al., 1993 [13]	Experimental animal study: immune complex induced inflammatory arthritis in mice	Knee	Single knee IA injection of liposomes encapsulating clodronate (6 µL containing 75 µg CL2MDP) vs. single knee IA injection with empty liposomes in control group.	2 groups of six mice	- Depletion (max at 7 days) and repopulation (60% at 30 days) of synovial lining cells (macrophages) but no effects on fibroblasts. - Reduction (max at 2 days) of proteoglycans synthesis and degradation. - Induced arthritis was prevented with CL2MDP-liposome
Van Lent et al., 1993 [14]	Experimental animal study: immune complex mediated inflammatory arthritis in mice	Knee	Single knee IA injection of liposomes encapsulating clodronate (6 µL containing 75 µg CL2MDP) vs. single knee IA injection with empty liposomes in control group.	2 groups of six mice	Depletion of phagocytic lining cells by clodronate in acute experimental arthritis resulted in lower inflammation and chemokine production (IL-1), cartilage damage, and joint swelling.
Van Lent et al., 1998 [15]	Experimental animal study: collagen-induced arthritis in mice	Knee	Single right knee IA injection of liposomes encapsulating clodronate (6 μL containing 30 μg CL2MDP) vs. single left knee IA injection with empty liposomes. The injections were done seven days before induced arthritis onset.	Groups of 10 mice	Clodronate reduces phagocytic lining cells number, decreasing cartilage destruction
Highton et al., 1999 [16]	Experimental animal study: antigen-induced arthritis model in sheep	Hock joint	Single right hock joint IA injection of liposomes encapsulating clodronate (0.5 mL containing 100 mg of clodronate), vs. single right hock joint IA injection of 0.5 mL of saline liposomes in control group vs. untreated group.	26 sheep (3 groups 10 + 10 + 6)	No significant differences among groups between intervention and control groups were found in histological and pathological analysis (lining layer thickness, degree of infiltration with mononuclear cells, fibrin deposition, neutrophils present or not).
Ceponiset al., 2001 [17]	Experimental animal study: antigen-induced arthritis in rabbits	Knee	Seven knee IA injections, once weekly, containing liposomes encapsulating clodronate (0.5 mL containing 0.145 mg of clodronate) vs. seven knee IA injections, once weekly, with empty liposomes in the control group	17 rabbits (clodronate group *n* = 10, control group *n* = 7)	Liposomal clodronate treated rabbits showed: - a reduction and delay in joint swelling; - a reduction in expression of matrix-bound TNFα, lining cell hyperplasia, and levels of RAM-11+ macrophages in the synovium; - prevention in cartilage proteoglycan loss. - lower radiological score at the end of weeks 2 and 4, but no longer present at 8 weeks.
Bakker et al., 2001 [18]	Experimental animal study: TGF-β induced arthritis in mice	Knee	Single knee IA injection of 6 µL liposome suspension containing 30 µg of clodronate.	Mice, *n*= N/A	Depletion of the lining cells, due to clodronate, resulted in a significant decrease in TGF-β- induced pathology. This finding was associated with a markedly reduced chondro-osteophyte formation and accumulation of extracellular matrix in synovium.
Blom et al., 2004 [19]	Experimental animal study: collagenase induced arthritis in mice	Knee	Single knee IA injection of 6 µL of clodronate liposome suspension (dosage not specified).	28 mice (22 treated, 6 untreated)	Intervention depletes synovial macrophages reducing osteophyte formation, fibrosis, and synovial activation, suggested by MRP8/14 expression. Moreover, intervention largely prevents the production of growth factors (TGFβ, BMP-2, and BMP-4) in superior layer lining cells of synovium.
Gomez-Barrena et al., 2006 [20]	Experimental animal study: antigen-induced arthritis in rabbits	Knee	Seven knee IA injections, once weekly, containing liposomes encapsulating clodronate (0.5 mL containing 0.145 mg of clodronate) vs. seven knee IA injections, once weekly, with empty liposomes in the control group	17 rabbits (10 treated, 7 control)	Clodronate enhances COMP levels into articular cartilage and reduces it in synovial tissue.
Blom et al., 2007 [21]	Experimental animal study: collagenase induced arthritis in mice	Knee	Single knee IA injection of liposomal encapsulated clodronate. (Dose not specified).	Mice (number not specified)	Intervention depletes macrophage resulting in complete inhibition of MMP-2, MMP-3, and MMP-9. This result was observed in synovium but not in cartilage tissue.
Sun et al., 2019 [22]	Experimental animal study: obesity model of OA	Knee	Three knee IA injections, one at week 1, one at week 2, and the last one at week 8, containing liposomes encapsulating clodronate (0.05 mg clodronate)	26 mice	Clodronate treated mouse showed: - a significant decreases in membrane thickness and influx of synoviocytes; - an increased content of proteoglycans; - a significant reduction in OA severity according to Mankin score; - a reduction in expression of COL10, DIPEN, and NITEGE.
Rossini et al., 2009 [23]	Multicentre randomized partially double-blind phase 2 study	Knee	First group: four knee IA injections, once weekly, containing 0.5 mg of clodronate; second group: four knee IA injections, once weekly, containing 1 mg of clodronate; third group: four knee IA injections, once weekly, containing 2 mg of clodronate; fourth group: four knee IA injections, twice weekly, containing 1 mg of clodronate; fifth group: four knee IA injections, once weekly, containing 20 mg of HA.	150 patients in 5 groups. Men and non-pregnant women aged 50–75 years with knee OA, radiographically confirmed with K-L grades of 2 or 3, symptomatic for at least 3 months.	Intervention groups show a significant (*p* < 0.001) reduction in the four VAS scores and Lequesne index. No significant difference among 5 groups was detected except for a dose–response relationship for active movement VAS pain outcome. Knee extension and mobility scores improved significantly at all time points in all treatment groups without statistical differences among groups.
Palmieri et al., 2013 [24]	Randomized double-blind study	Knee	Group one: single knee IA injection of HA alone (66 mg/2 mL) into each knee; second group: single knee IA injection of HA (49.5 mg/1.5 mL) plus diclofenac sodium (5 mg) into each knee; third group: single knee IA injection of HA (49.5 mg/1.5 mL) plus sodium clodronate (5 mg/0.5 mL) into each knee. Moreover, 0.5 mL of 1% lidocaine was added to every injection.	62 patients in three groups (20/21/21). Patients with symptomatic bilateral medial tibiofemoral knee OA (K-L grade II and III) and pain in both knees (VAS > 30) in the month before	In the three groups of patients, there was a significant reduction in mean VAS pain score, ESR, and CRP levels. The combination with sodium clodronate was the most beneficial in terms of improvement in VAS pain score (10.1% improvement vs. group 1; 8.8% improvement vs. group 2).
Rossini et al., 2015 [10]	Double-blind phase 3 randomized clinical trial.	Knee	Four knee IA injections, once weekly, containing 2 mg of clodronate vs. four knee IA injections, once weekly, containing placebo.	80 patients in 2 groups (40/40). Patients between 50 and 75 years, affected by KOA defined according to ACR criteria, radiographically confirmed with a K-L scale ≥ 2, symptomatic for at least 3 months (VAS > 40).	Intervention shows a significant reduction in VAS pain after 5 weeks from last injection (−114.6 vs. −87.2; *p* < 0.05). Better results were found for Lequesne index, global KOA evaluation, and the WOMAC pain subscale.

**Acronyms:** CL2MDP: dichloromethylene diphosphonate; IL1: interleukin 1; PMNs: polymorphonuclear cells; CIA: collagen type II arthritis; MRP: migration inhibitory factor-related proteins; TNFα: tumor necrotic factor alfa; BMP-2/-4: bone morphogenetic protein; COMP: cartilage oligomeric matrix protein; AIA: antigen-induced arthritis; OA: osteoarthritis; MMP-2/-3/-9: matrix metalloproteinase; VAS: visual analogic scale; IA: intra-articular; HA: hyaluronic acid; ESR: erythrocyte sedimentation rate; CRP: C-reactive protein; WOMAC: Western Ontario and Mc Master University. K-L: Kellgren–Lawrence; ACR: American College of Rheumatology; COL10: collagen type 10; NITEGE: the aggrecanase-generated C-terminal neoepitope; DIPEN: MMP-generated C-terminal neoepitope.

## Data Availability

Not applicable.

## Abbreviations

IA	Intra-articular
OA	Osteoarthritis
CL2MDP	dichloromethylene diphosphonate
IL1	interleukin 1
PMNs	polymorphonuclear cells
CIA	collagen type II arthritis
MRP	migration inhibitory factor-related proteins
TNFα	tumor necrotic factor alfa
BMP-2/-4	bone morphogenetic protein
COMP	cartilage oligomeric matrix protein
AIA	antigen-induced arthritis
MMP-2/-3/-9	matrix metalloproteinase
VAS	visual analogic scale
HA	hyaluronic acid
ESR	erythrocyte sedimentation rate
CRP	C-reactive protein
WOMAC	Western Ontario and McMaster University
K-L	Kellgren–Lawrence
ACR	American College of Rheumatology
COL10	collagen type 10
